# Summary of the National Advisory Committee on Immunization (NACI) Statement on the Prevention of Respiratory Syncytial Virus (RSV) in Infants

**DOI:** 10.14745/ccdr.v51i04a01

**Published:** 2025-04-03

**Authors:** April Killikelly, Winnie Siu, Nicholas Brousseau

**Affiliations:** 1 Public Health Agency of Canada, Centre for Immunization and Respiratory Infectious Diseases, Ottawa, ON; 2 School of Epidemiology and Public Health, Faculty of Medicine, University of Ottawa, Ottawa, ON; 3 Département de médecine sociale et préventive, Faculté de médecine, Université Laval, Québec, QC

**Keywords:** National Advisory Committee on Immunization, RSV, Canada, infants, nirsevimab/BEYFORTUS, RSVpreF/ABRYSVO, vaccine guidance, passive immunization, monoclonal antibody

## Abstract

**Background:**

Immunization programs for the prevention of respiratory syncytial virus (RSV) in infants have been available in Canada since the authorization of palivizumab in 2002. However, these programs have been limited to only those infants at highest risk for severe RSV disease. The authorization of new passive immunizing products to prevent RSV, including a new monoclonal antibody (nirsevimab) and a vaccine administered in pregnancy (RSV pre-fusion stabilized F protein; RSVpreF) offers the opportunity to prevent RSV in more Canadian infants. The objective of this article is to summarize guidance from the National Advisory Committee on Immunization (NACI) on the prevention of RSV in infants.

**Methods:**

NACI established key policy questions and performed an evidence review and synthesis. NACI made evidence-based recommendations in consideration of the burden of illness to be prevented, safety and efficacy of the new immunizing products, economic evidence and ethics, equity, feasibility, and acceptability.

**Results:**

Nirsevimab and RSVpreF offer protection against severe outcomes of RSV disease, including hospitalization and intensive care unit admission. Nirsevimab protection may be slightly higher and may last longer than protection offered by RSVpreF. Nirsevimab and RSVpreF also have a similar frequency of adverse reactions for both pregnant and infant participants. The RSVpreF vaccine may increase the risk of severe local adverse events compared to placebo for pregnant recipients. In RSVpreF clinical trials, an imbalance was observed in late preterm birth between RSVpreF and placebo recipients. It is unclear whether there is a causal relation with the vaccine as the currently available data is inconclusive.

**Conclusion:**

Based on new evidence, NACI recommends building towards a universal RSV immunization program for all infants. Currently, nirsevimab is preferred over RSVpreF. Program introduction could occur in stages depending on access to supply, cost effectiveness, and affordability of available options.

## Introduction

Respiratory syncytial virus (RSV) is one of the most common respiratory viruses in infants and young children, infecting almost all by the age of two years old. Respiratory syncytial virus can cause serious respiratory disease in infants, young children and older adults. Approximately 1%–2% of infants will be hospitalized during their first year of life ((1)). Respiratory syncytial virus has a seasonal pattern of activity, where infections are usually more common in the winter, with variation in the timing and magnitude of the peak. Prior to the COVID-19 pandemic, the RSV season in most of Canada was typically November to April, but this may vary by region.

Severe RSV disease is most common in young infants in their first months of life. Although the risk of severe RSV disease is higher in infants with certain medical conditions, including prematurity, term infants account for the highest number of infants with severe RSV disease. Infants with certain medical conditions remain at risk for severe RSV disease during their second RSV season.

Immunization programs for the prevention of RSV in infants have been available in Canada since the authorization of palivizumab in 2002 ((2,3)); however, these programs are limited to only those infants at highest risk for severe RSV disease. Health Canada has recently authorized two immunization products, both based on the pre-fusion stabilized F protein (preF) from RSV, to protect infants from RSV using passive immunity. Nirsevimab (BEYFORTUS^TM^, Sanofi) is a monoclonal antibody authorized with an indication to directly protect all infants in their first RSV season and children who remain vulnerable to severe RSV disease in their second RSV season. The RSVpreF (ABRYSVO^TM^, Pfizer) vaccine is authorized with an indication to protect infants in their first RSV season through the passive transfer of maternal antibodies to the fetus by active immunization of a pregnant woman or pregnant person at 32–36 weeks of gestation (wGA).

The National Advisory Committee on Immunization (NACI) provides the Public Health Agency of Canada (PHAC) with recommendations ((4)) regarding the use of vaccines and immunization products for RSV, which reflect the latest evidence for RSV epidemiology, immunization practices, and product authorization and availability in Canada. Recommendations also take into account ethics, equity, feasibility and acceptability considerations, and economic analysis. NACI developed updated advice on the prevention of RSV in infants, which was triggered by the authorization of new products to protect infants from RSV. This work was led by the RSV working group (RSV WG) and involved a thorough review and evaluation of the literature, as well as discussion and debate at the scientific and clinical practice levels.

## Methods

The NACI RSV WG reviewed key questions and performed evidence reviews and syntheses. In consideration of the burden of illness to be prevented, the burden of disease, safety, efficacy, ethics, equity, acceptability, feasibility and economics, the RSV WG proposed recommendations for vaccine use to NACI. All evidence was reviewed according to the Grading of Recommendations, Assessment, Development, and Evaluation (GRADE) methodology and summarized in evidence tables. NACI approved specific evidence-based recommendations and summarized the rationale and relevant considerations in the statement.

## Results

### Efficacy

The evidence derived from three randomized controlled trials (RCTs) ((5–8)) suggests that nirsevimab results in a reduction in intensive care unit (ICU) admission associated with RSV, hospitalization associated with RSV, and medically attended RSV respiratory tract infection (RTI) in infants entering their first RSV season. In infants considered at high risk entering their first and second RSV season, the evidence suggests that nirsevimab likely results in a reduction in ICU admission associated with RSV, hospitalization associated with RSV and medically attended RSV RTI compared to palivizumab. However, there was limited evidence on the effect of nirsevimab against death due to RSV in infants.

The evidence from two RCTs ((9,10)) suggests that RSVpreF vaccine administered to pregnant women and pregnant people results in a reduction in ICU admission associated with RSV, hospitalization associated with RSV and medically attended RSV RTI in infants entering their first RSV season. However, there was limited evidence available on the effect of RSVpreF vaccine against death due to RSV in infants. No data are available on either the efficacy or safety of additional doses of RSVpreF given during subsequent pregnancies.

Compared to palivizumab, nirsevimab has an extended half-life and comparable, or potentially better, protective efficacy (when nirsevimab was compared to palivizumab, relative vaccine effectiveness [VE] was 53%; [95% CI: −279%–94%] at 150 days for RSV RTI with hospitalization). In addition, the increase in predicted half-life may mean once-per-season dosing of nirsevimab compared to monthly dosing for palivizumab.

Compared to RSVpreF, nirsevimab may be slightly more effective and may offer a longer duration of protection. The review of available evidence from clinical trials demonstrated that RSVpreF is effective at preventing severe RSV disease in an infant during the first months of life (VE of 57%; 99.17% CI: 10%–81% at 180 days for RSV RTI with hospitalization). The available data for nirsevimab demonstrate a higher vaccine efficacy for potentially a longer period (VE of 81%; 95% CI: 64%–91% at 150 days for RSV RTI with hospitalization).

### Safety

The evidence suggests that among infants entering their first RSV season, nirsevimab is not likely to increase the risk of severe systemic and local adverse events (AEs) compared to placebo. In infants considered at high risk entering their first and second RSV season, nirsevimab is not likely to increase the risk of severe systemic and local AEs when compared to palivizumab. Moreover, no meaningful differences in serious AEs were observed when nirsevimab was compared to a placebo or to palivizumab.

Among pregnant women and pregnant people, RSVpreF vaccine may not result in an increase in severe systemic AEs, but may increase the risk of severe local AEs compared to placebo. With respect to any potential effects on the fetus, when RSVpreF is administered in pregnancy, receipt does not result in an increase in severe systemic AEs compared to placebo among infants entering their first RSV season. When RSVpreF is administered in pregnancy, the frequency of serious AEs was similar in pregnant women, as well as in infant participants across the RSVpreF and placebo recipients. However, an imbalance was observed in preterm births between RSVpreF and placebo recipients. It is unclear whether there is a causal relation with the vaccine, as the currently available data are inconclusive. Limiting vaccine administration to the Health Canada approved dosing intervals from 32 through 36 wGA will mitigate potential risk of preterm birth. NACI continues to monitor the RSVpreF vaccine safety data as they emerge and will update its recommendation if needed.

### Ethics, equity, feasibility, acceptability

When interpreting the epidemiological trends to inform the recommendations, equity considerations include acknowledging that available evidence for some populations is limited and may be biased, for example, due to systemic limitations in available data for racialized groups. Consideration should be given for diverse contexts of equity-implicated communities. One example where diverse contexts may apply is Indigenous groups across various settings (e.g., urban, rural, on reserve, off reserve). Complex medical transport for RSV care can have a disproportionate impact on infant health and can create community disruption. Complex transport settings include situations where transport distance may be very long (e.g., ground ambulance transport over several hours), but also may include shorter distances that require air or other complex transport or strategies.

### Economics

Systematic reviews ((11)) and a *de novo* model-based economic evaluation ((12)) were conducted by the NACI Secretariat to support decision-making for the use of nirsevimab and RSVpreF for prevention of RSV in infants. The model-based economic analyses showed that an all-infant program for nirsevimab and an RSVpreF program for all pregnant women and pregnant people were not cost-effective at commonly used cost-effectiveness thresholds, even with modelled longer duration of protection. However, programs that limit nirsevimab use to those at increased medical risk due to RSV (defined as prematurity less than or equal to 36^6/7^ wGA in the economic analysis) or living in settings with a higher RSV hospitalization rate and healthcare costs, were considered cost-effective at a cost-effectiveness threshold of $50,000 per quality-adjusted life year (QALY) gained. RSVpreF for all pregnant women and pregnant people combined with a high-risk program for nirsevimab may be cost-effective in settings with higher RSV hospitalization rates and healthcare costs.

## Discussion

### Recommendations

Considering the significant burden of disease in all infants from RSV and the impacts of RSV on the Canadian health system, NACI recommends building towards a universal RSV immunization program for all infants. Currently, nirsevimab is preferred over palivizumab and RSVpreF. Program introduction could occur in stages depending on access to supply, cost effectiveness and affordability of available options.

NACI recommends RSV immunization programs use nirsevimab to prevent severe RSV disease. Programs can build and expand over time depending on access to supply, cost effectiveness, and affordability of available options. Nirsevimab should be prioritized for infants as described below.

### Priority 1

• Entering, or born during, their first RSV season who are at increased risk of severe RSV disease, including those who are born at less than 37 wGA (**Table 1**).

**Table 1:** Definition of infants at increased risk of severe RSV disease

Term	Definition
Infants at increased risk of severe RSV disease during their first RSV season	All premature infants (i.e., born less than 37 wGA) Chronic lung disease, including bronchopulmonary dysplasia, requiring ongoing assisted ventilation, oxygen therapy or chronic medical therapy in the six months prior to the start of the RSV season Cystic fibrosis with respiratory involvement and/or growth delay Haemodynamically significant chronic cardiac disease Severe immunodeficiency Severe congenital airway anomalies impairing clearing of respiratory secretions Neuromuscular disease impairing clearing of respiratory secretions Down syndrome
Infants at ongoing increased risk of severe RSV disease during their second RSV season	All those listed above, except for infants born at less than 37 wGA and infants with Down syndrome who do not have another medical condition on the list

Abbreviations: RSV, respiratory syncytial virus; wGA, weeks of gestation

• Entering their second RSV season and at ongoing increased risk of severe RSV disease (Table 1).

• Entering, or born during, their first RSV season, whose transportation for severe RSV disease treatment is complex, and/or whose risk of severe RSV disease intersects with established social and structural health determinants, such as those experienced by some Indigenous communities across First Nations, Métis and Inuit populations.

### Priority 2

• If nirsevimab is priced in a manner to make such programs cost effective, NACI recommends nirsevimab be considered for any infant less than 8 months of age entering, or born during, their first RSV season, through universal immunization programs to prevent severe RSV disease.

NACI recommends RSVpreF may be considered as an individual decision by a pregnant woman or pregnant person together with information from their pregnancy care provider, in advance of, or during, the RSV season, to prevent severe RSV disease in their infant. At the present time, NACI does not recommend an immunization program for RSVpreF. More data and information are expected to emerge over time and NACI will reconsider this recommendation in the future.

For administration of RSVpreF, consideration should be given to gestational timing and the start of the RSV season. For example, RSVpreF could be administered starting in September to protect infants expected to be born during the RSV season in November, provided that gestational age is 32 weeks or greater at time of vaccination.

Indigenous peoples experience a high burden of illness due to social, environmental and economic factors, rooted in the history of colonization and systemic racism (i.e., structural inequity); this recommendation for nirsevimab for Indigenous infants aims to address the severe health inequities that exist and prioritize an intervention for people who have historically been, and continue to be, marginalized. Complex medical transport for RSV care can have a disproportionate impact on infant health and can create community disruption. Complex transport settings include situations where transport distance may be very long (e.g., ground ambulance transport over several hours), but also may include shorter distances that require air or other complex transport or other complex strategies.

## Limitations

Jurisdictions are encouraged to define the RSV season and administer nirsevimab based on local epidemiology.

## Conclusion

For the prevention of RSV in infants, NACI recommends building towards a universal RSV immunization program for all infants. Program introduction could occur in stages depending on access to supply, cost effectiveness and affordability of available options. Nirsevimab is preferred over palivizumab. In contexts where there is limited or no availability of nirsevimab, palivizumab should be used according to the NACI 2022 recommendations ((3)). Nirsevimab is also preferred over RSVpreF. If it is anticipated that nirsevimab will be administered to a healthy infant, then RSVpreF in pregnancy may not provide added benefit for the healthy infant. NACI will review the available evidence and recommendations when there are new data and/or product indications.
